# The use of a microcomputer system for peak recognition, data processing and representation in continuous-flow analysis

**DOI:** 10.1155/S1463924683000073

**Published:** 1983

**Authors:** H. Baadenhuijsen, Th. Zelders

**Affiliations:** 1 Department of Internal Medicine Division of Clinical Chemistry St. Radboud University Hospital Geert Grooteplein Zuid 8 Nijmegen The Netherlands; 2 Department of Instrumentation Faculty of Medicine University of Nijmegen F.C. Donderslaan 2 Nijmegen The Netherlands


Journal of Automatic Chemistry, Volume 5, Number 1 (January–March 1983), pages 18–21

### The use of a microcomputer system for peak recognition, data processing and representation in continuous-flow analysis

**H. Baadenhuijsen**
*Department of Internal Medicine, Division of Clinical Chemistry, St. Radboud University Hospital, Geert Grooteplein Zuid 8, Nijmegen, The Netherlands*

**and Th. Zelders**
*Department of Instrumentation, Faculty of Medicine, University of Nijmegen, F.C. Donderslaan 2, Nijmegen, The Netherlands*

### Introduction

Confronted with an increasing laboratory work-load, a device which would be able to eliminate such tedious tasks as the manual read-out procedure of the analyser-recorder output with peak-height tracings of calibration solutions and patient serum specimens was sought. Apart from the amount of work in their laboratory the authors were also concerned about a small, but significant, number of clerical errors which seemed to be related to the work-load. Before setting up a totally integrated computer-based on-line data acquisition system, a stand-alone read-out interface for one of the busiest continuous-flow channels (serum urea measured according to the method of Jung *et al.*) was developed to cope with immediate problems.

In order that the properties of the interface reported in this paper can be properly appreciated, a short description of the features of the authors' laboratory's continuous-flow procedures follows. The configuration is composed of modular hardware made by Breda Scientific, The Netherlands. The system runs in the so-called 'Autoanalyzer I' mode, which means that after every sample the system is rinsed long enough to almost return to the base-line signal level before the next sample is sensed. Peaked signal levels account for about 80–90% of the maximum signal plateau [2]. The chemistry is calibrated by means of five calibration standard solutions run after a maximum of 13 patient samples. The standard solution series is bracketed within two additional sample cups of rinse solution (9 g/l of sodium chloride).

In order to be as flexible as possible, it was necessary for the interface to meet the following requirements.

(1) It needed to be easily reprogrammable to other chemistries, which would be carried out by relatively inexperienced laboratory technicians.
(2) There should be no restrictions on the run length.
(3) A real on-line read-out was needed in order to offer immediate evaluation of stat sample requests.
(4) It should be possible to freely enter time settings of the sample and rinse frequency as necessary.
(5) The flow system should be continuously monitored for frequency deviations.
(6) The value of the colorimeter signal should be continuously displayed digitally.
(7) The results of each run, together with some basic statistics, should be recorded for future use—for statistical analysis and research.
(8) The system should offer a software-controlled stop-monitoring capability.

A complete listing of the programs is available upon request from the authors.

### Hardware

The system (figure 1) was configured around an ITT 2020 with 48 kbytes of memory, equipped with a 9 in monitor, a floppy disc drive (Apple II) and a matrix printer (Epson TX-80). The ITT 2020 also has an Autostart ROM, a printer interface (Epson, slot 1), an interrupt timer (CCS 7440A, slot 2), an analogue/digital (A/D) converter (CCS 7470A, slot 3), an amplifier board (home-made, slot 4) and a disc drive controller (Apple II, slot 6).

### Logical design

The sampling of the colorimeter signal is based on the recognition of the typical rise-and-fall pattern of a continuous-flow peak. After the power is turned on, the autostart ROM loads and runs the program, which is written in BASIC. The program starts verifying some basic variables: for example date, frequency and time between two successive specimens. The latter two variables are necessary to construct the time window in which the peaks have to be obtained. Then the interrupt-timer (programmable in multiples of 0·1 s intervals, but used for samples with a 2 s interval cycle) is started and the A/D converter is triggered for its first conversion. The A/D converter is instructed to dump all sampled signals into data memory locations 4000H–8000H; this is done through a routine which

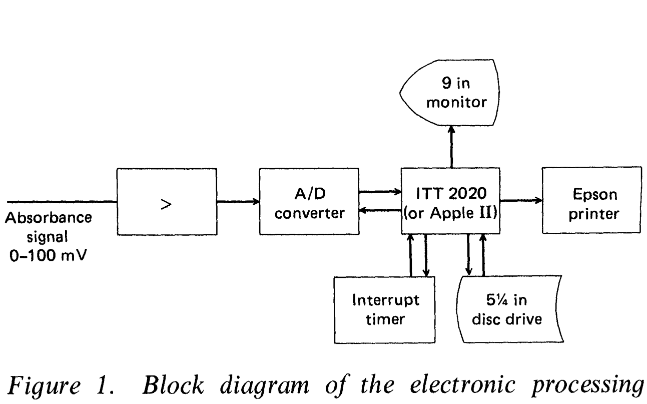

*Figure 1. Block diagram of the electronic processing system.*

was written in 6502 Assembler. In order to obtain a correct starting base-line signal level, the program judges the data samples in memory to be steady within certain limits for at least 15 samples; this scanning is carried out with a series of PEEK statements. Although the authors were aware of the existence of a number of sophisticated peak-picking procedures [3–5], they decided to implement a very simple and effective procedure.

The subroutine that recognizes each recorded analyser peak is based on an algorithm which analyses the previous 15 electrical signal samples scanned. During this process, the moving averages of the first five and the last five points of these 15 samples are continuously compared. Location of the peak maximum agrees with that point, in the midst of the moving window, where for the first time the average of the last five

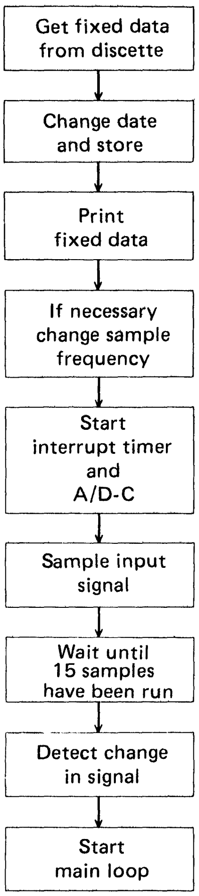

*Figure 2 (a). Flowchart for initiating the main program.*

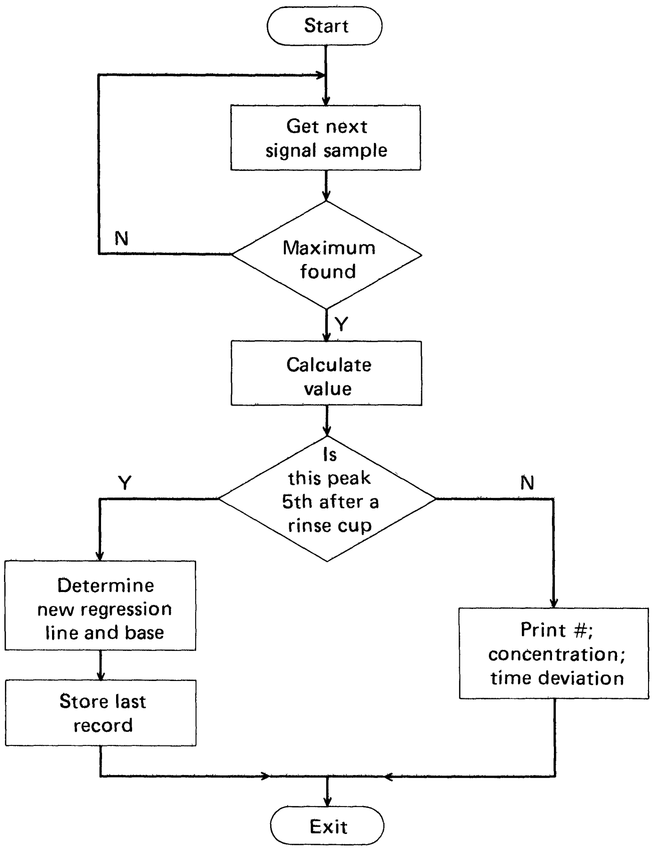

*Figure 2 (c). Program flow for peak evaluation.*

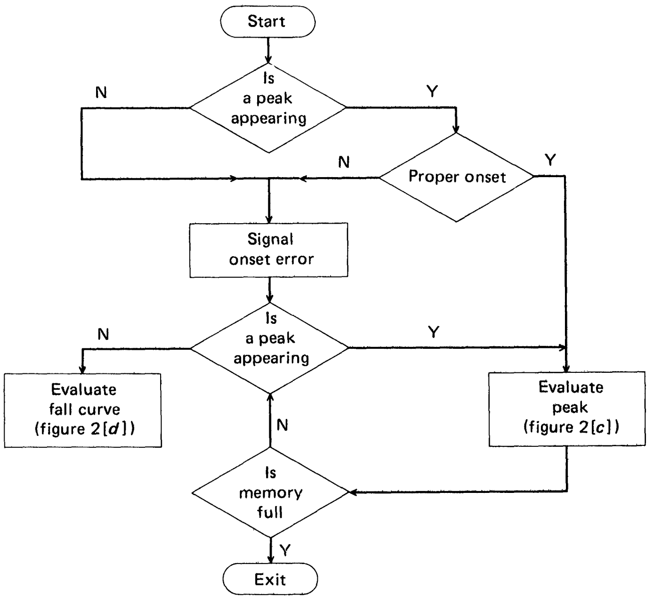

*Figure 2 (b). Program flow main loop.*

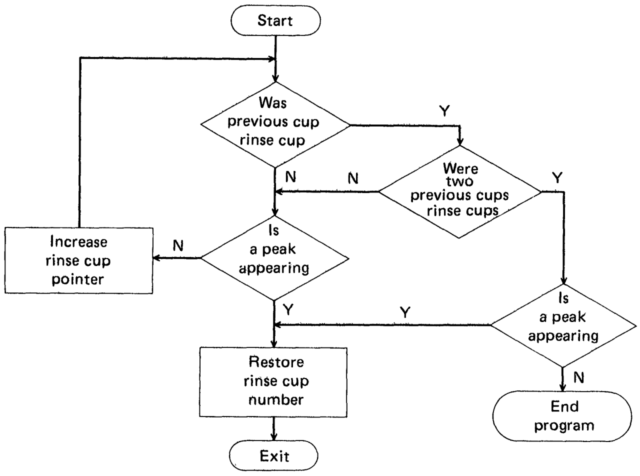

*Figure 2 (d). Program flow for evaluation of fall curve.*

samples is less than the average of the first five samples. So the averaged value of sample Nos. 6, 7 and 8 is assumed to represent the value of the maximum output. Together with the sample cup number, and the deviation in time of the pre-estimated location of the maximum, this value is printed out, after some calculation, as a concentration value. This concentration value is calculated by substituting the peak value in the regression equation obtained for the run in question. The system is required to look for peak values and locates the insertion of extra cups with rinse solution when no peaks occur within the time window. If three successive rinse cups occur the computer will stop the program and alert the technician with a printed message. The computer analyses every five peaks following a rinse cup looking for a calibration series. A calibration series is recognized as such when the linear regression coefficient of the series of five peaks of interest equals or surpasses 0·995. When this coefficient lies between 0·98 and 0·995 the computer looks for the point which contributes most to the observed deviation of linearity; after eliminating the point, the line of best fit is again constructed with the four remaining points. When this procedure results in an acceptable linear fit, the program continues with this new regression equation, printing an 'alert' to the effect that one calibration point has been omitted from the calculation. As soon as a new calibration series has been detected, the computer looks for the presence of one extra rinse cup in order to establish the new base-line signal level. All the peaks between two successive calibration series are stored as one new record on floppy disc. The former value and new value of the slope, the base-line signal level and the regression coefficient are printed out. A flowchart describing the program is shown in figure 2.

### Additional features

The last series of observations can be printed out by pressing the 'S' key whenever required.

If the program flow has been stopped or interrupted for any reason (by striking the 'Ctrl-Z' or 'Ctrl-C' key), the timer and the A/D converter still continue to dump their signal samples in the memory locations 4000H–8000H. The stored samples are processed from the start by a program called 'CATCH' at an increased speed, until the data processing is synchronized with data acquisition.

The authors still use a recorder hard-copy output of the peak tracings for back up, but the system also has a plot feature. The stored signals can be graphically visualized by running the auxiliary program 'PLOT', which shows the first or the second half of the memory contents on the monitor.

As already mentioned, all results between two successive calibration series are stored as one record on disc, together with related data such as slope, intercept and regression coefficient. These records can be processed with a separate program called 'READRECORD' for later retrieval. The total capacity of such records is about 100 records/disc.

```
DATE: JUL-9

STANDARDS

3

6

9

12

15

DETERM.

UREA

 #        CONC.     DEV.

 1         3.01     135
 2         6.08       0
 3         9.15      -1
 4        11.98       0
 5        15.06       0

OLD SLOPE  43       OLD BASIS  18

NEW SLOPE  44.58    NEW BASIS  26

CORR. COEFF.: .9998  REC. #:  2

 6          .02       0  RINSE CUP
 7        12.75      -2
 8         9.91       0
 9         8.18       0
10         5.07      -1
11        31.09       0
12        18.17       0
13         5.77       0
14        13.73       0
15         7.39      -1
16         4.68       0
17        11.01       1
18         5.21       0
19         5.98      -1
20          .06       0  RINSE CUP
21         3.09      -1
22         6.10       0
23         9.12      -1
24        12.10       0
25        15.20      -1
```

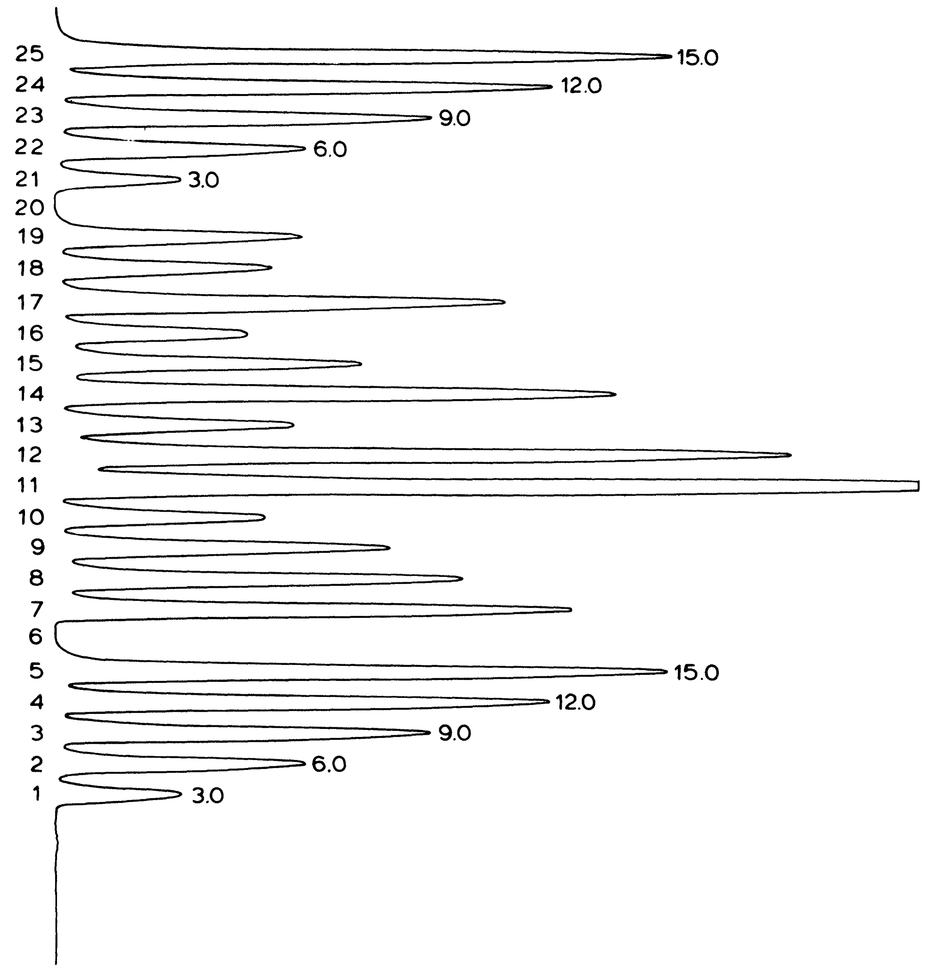

*Figure 3. Example of the printer and recorder output. (Note that although for patient sample 5 [peak 11] the recorder tracing is far beyond the full scale limit, the Lambert-Beer Law holds for much higher concentrations; the detector output signal was evaluated as being equivalent to a concentration of 31·1 mol/l, which is in close agreement with the value of peak 18, being the same sample diluted six times. Peak 19 is sample 12 diluted three times.)*

### Results and discussion

The system described has been in daily use for about 18 months. Figure 3 shows an example of the printer and recorder output for a representative sample run. Due to an absence of restrictions on run length and on the location of the calibration series within a particular run, the system has been proven very flexible. In spite of the relatively wet and dusty work environment, only two occasions of floppy disc deterioration were discovered in the 18-month period. When setting up the logical design, it was realized that it was theoretically possible that in the extremely unlikely situation that a series of five consecutive patient samples would have a concentration sequence resembling the standard calibration series, they would be recognized and processed as a calibration series. This risk was removed by checking on the precedence of the standards with a rinse cup. Only once was a situation where five consecutive patient samples were ascribed a correlation coefficient higher than 0·995 actually encountered.

After having analysed about 15 000 serum urea samples it can be reasonably concluded that this personal computer system is able to fulfill exacting demands as a measuring and controlling device for a continuous-flow analyser.

### References

1. Jung, D., Biggs, H., Erikson, J. and Ledyard, P. V., *Clinical Chemistry*, **21** (1975), 1136.

2. Jansen, A. P., Peters, K. A. and Zelders, T., *Clinica Chimica Acta*, **27** (1970), 125.

3. Goedert, M. and Guiochon, G., *Journal of Chromatographic Science*, **11** (1973), 326.

4. Wijtvliet, J. J. M. (Thesis, T. H. Eindhoven, The Netherlands, 1972).

5. Subroutine PEAK from Digital Equipment Corporation: Laboratory Subroutine Package AA-C984B-TC.

### COURSE ANNOUNCEMENT

*UMIST/Sira practical course on fibre optics for instrumentation: basics, sensors and systems*

To meet continuing demand, the UMIST/Sira course on fibre optics for instrumentation will be repeated on 28–30 June 1983 at UMIST, Manchester, UK. This three-day training course consists of lectures, demonstrations, practical sessions and discussion groups. The aim is to provide participants with a basic knowledge of fibre optics and an appreciation of the technical and commercial potential of fibre optics as applied to instrumentation.

The course is intended for potential users of fibre optics in conjunction with instrumentation. Participants need not have a detailed prior knowledge of fibre optics.

The lectures, in conjunction with the demonstrations and practical exercises, cover the following topics:

Basic optics and principles of fibre optics
Optical radiation sources and detectors
Optical effects of potential use in measurement
Fibre optic cables
Couplers, connectors and splices
Optical sensors, with case studies
Systems design
Fibre optic connectors between instruments
Economics of fibre optic systems
The future for fibre optics in instrumentation.

*The course costs £345, which includes three days' accommodation and meals. Details from Sira's Conference Unit at South Hill, Chislehurst, Kent BR7 5EH, UK. Tel. 01 467 2636.*

### SYMPOSIUM

*International Symposium on Chromatography and Mass Spectrometry in Nutrition Science and Food Safety*

The meeting, which is to be held at the Hotel Eden au Lac in Montreux, Switzerland, from 20 to 22 June 1983, is being arranged by the Italian Group for Mass Spectrometry in Biochemistry and Medicine together with Nestlé Products Technical Assistance Company Ltd. All the latest aspects of chromatography and mass spectrometry in nutrition science and food safety are intended to be illustrated and discussed. Main topics are: food science, flavours and aromas, nutritional biochemistry in humans and animals, disease in relation to nutrition, food safety, and improvements in the methodology of chromatography and mass spectrometry in nutrition science and food safety. The Chairmen are Drs Alberto Frigerio (Italian Group for Mass Spectrometry in Biochemistry and Medicine) and Hubert Milon (Nestlé Products Technical Assistance Company Ltd).

*Further information from the Italian Group for Mass Spectrometry in Biochemistry and Medicine, Via Eritrea 62, I 20157 Milan, Italy.*

